# Supplementary material for: Fabrication and Characterization of Zein Composite Particles Coated by Caseinate-Pectin Electrostatic Complexes with Improved Structural Stability in Acidic Aqueous Environments
Source: Molecules. 2019 Jul 11;24(14):2535. doi: 10.3390/molecules24142535 (PMC6681193; doi:10.3390/molecules24142535)
Supplement: Supplementary file 1 [file molecules-24-02535-s001.pdf]

Fabrication and characterization of zein composite particles coated by caseinate-pectin electrostatic complexes with improved structural stability in acidic aqueous environment

Yaqiong Zhang [1], Bo Wang [1], Yan Wu [1], Boyan Gao [1,*], Liangli (Lucy) Yu [2]

[1] *Department of Food Science & Engineering, School of Agriculture and Biology, Shanghai Jiao Tong University, Shanghai 200240, China*

[2] *Department of Nutrition and Food Science, University of Maryland, College Park, MD 20742, United States*

* To whom correspondence should be addressed:

Boyan Gao, Ph. D.

Tel.: 86-021-34204538; E-mail: gaoboyan@sjtu.edu.cn

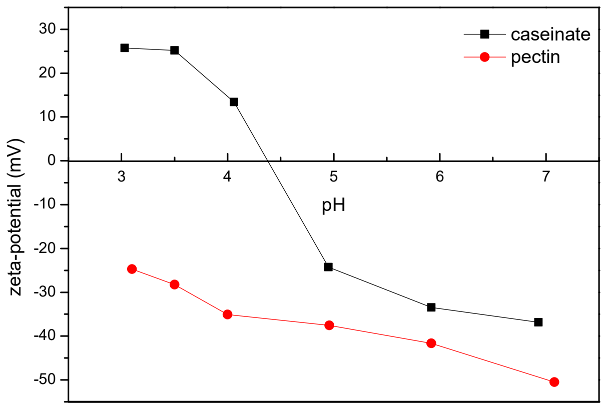

Figure S1. ζ-potential as a function of pH of caseinate (■) and pectin (●) aqueous solutions. The concentration of caseinate and pectin is 1 mg/mL, respectively.

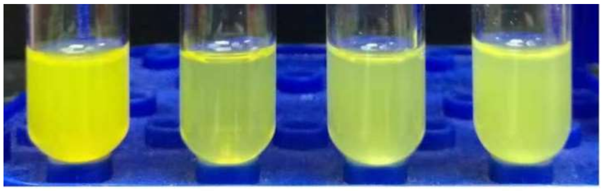

Figure S2. Visual appearance of the freshly-prepared Cur-loaded zein-caseinate-pectin particle dispersions in water.
